# Biomarkers for antitumor activity of bevacizumab in gastric cancer models

**DOI:** 10.1186/1471-2407-12-37

**Published:** 2012-01-25

**Authors:** Yoriko Yamashita-Kashima, Kaori Fujimoto-Ouchi, Keigo Yorozu, Mitsue Kurasawa, Mieko Yanagisawa, Hideyuki Yasuno, Kazushige Mori

**Affiliations:** 1 Product Research Department, Chugai Pharmaceutical Co., Ltd., 200, Kajiwara, Kamakura, Kanagawa, Japan 247-8530

## Abstract

**Background:**

Bevacizumab is a humanized monoclonal antibody to human vascular endothelial cell growth factor (VEGF) and has been used for many types of cancers such as colorectal cancer, non-small cell lung cancer, breast cancer, and glioblastoma. Bevacizumab might be effective against gastric cancer, because VEGF has been reported to be involved in the development of gastric cancer as well as other cancers. On the other hand, there are no established biomarkers to predict the bevacizumab efficacy in spite of clinical needs. Therefore, we tried to identify the predictive markers for efficacy of bevacizumab in gastric cancer patients by using bevacizumab-sensitive and insensitive tumor models.

**Methods:**

Nine human gastric and two colorectal cancer mouse xenografts were examined for their sensitivity to bevacizumab. We examined expression levels of angiogenic factors by ELISA, bioactivity of VEGF by phosphorylation of VEGFR2 in HUVEC after addition of tumor homogenate, tumor microvessel density by CD31-immunostaining, and polymorphisms of the VEGF gene by HybriProbe™ assay.

**Results:**

Of the 9 human gastric cancer xenograft models used, GXF97, MKN-45, MKN-28, 4-1ST, SC-08-JCK, and SC-09-JCK were bevacizumab-sensitive, whereas SCH, SC-10-JCK, and NCI-N87 were insensitive. The sensitivity of the gastric cancer model to bevacizumab was not related to histological type or HER2 status. All tumors with high levels of VEGF were bevacizumab-sensitive except for one, SC-10-JCK, which had high levels of VEGF. The reason for the refractoriness was non-bioactivity on the phosphorylation of VEGFR2 and micro-vessel formation of VEGF, but was not explained by the VEGF allele or VEGF165b. We also examined the expression levels of other angiogenic factors in the 11 gastrointestinal tumor tissues. In the refractory models including SC-10-JCK, tumor levels of another angiogenic factor, bFGF, were relatively high. The VEGF/bFGF ratio correlated more closely with sensitivity to bevacizumab than with the VEGF level.

**Conclusions:**

VEGF levels and VEGF/bFGF ratios in tumors were related to bevacizumab sensitivity of the xenografts tested. Further clinical investigation into useful predictive markers for bevacizumab sensitivity is warranted.

## Background

Vascular endothelial growth factor (VEGF), a diffusible glycoprotein produced by normal and neoplastic cells, is an important regulator of physiologic and pathologic angiogenesis. Increased VEGF levels in serum or tumor tissue have been reported to correlate with poor survival; therefore, efficacy of anti-VEGF therapy is expected in clinical application [1,2].

Bevacizumab is a humanized monoclonal antibody to human VEGF that inhibits VEGF-mediated angiogenesis in many types of tumors. In the US and EU, bevacizumab is used in combination with standard chemotherapies for patients with colorectal cancer, non-small cell lung cancer, breast cancer, and glioblastoma. Although bevacizumab improves progression-free survival (PFS) in these cancers, it is not effective for all patients. Predictive markers of bevacizumab efficacy have been assessed in many clinical trials [3], however, no validated biomarker is available to predict bevacizumab efficacy and identify the patients who could benefit from bevacizumab. Therefore, it is important to investigate the biomarker of bevacizumab efficacy from the phase of clinical development for other cancer types.

Gastric cancer is one of the most malignant cancers and second leading cause of cancer death in the world [4]. The incidence is reportedly highest in Asia, South America, and Southern Europe [5]. Increased levels of VEGF expression have been found in gastric cancers as well as in tumors of lung, breast, thyroid, gastrointestinal tract, kidney, bladder, ovary, cervix, and pancreas, angiosarcomas and glioblastomas [6,7]. A previous report suggests the possibility of VEGF as a prognostic factor of gastric cancer [8]. Therefore, bevacizumab may also be effective against gastric cancers [9]. In the present study, the relationship between the efficacy of bevacizumab, selected biomarkers in gastric cancers and various histological types of gastric cancer has been examined.

## Methods

### Test agents

Bevacizumab was provided by F. Hoffman-La Roche (Nutley, NJ, USA) as a liquid and diluted with saline. Human immunoglobulin G (HuIgG) was purchased from MP Biomedicals, Inc. (Aurora, OH, USA) and was reconstituted with water and diluted with saline.

### Animals

Male 5-week-old BALB/c-nu/nu mice (CAnN.Cg-Foxn1 < nu >/CrlCrlj nu/nu) were obtained from Charles River Japan (Yokohama, Japan). All animals were allowed to acclimatize and recover from shipping-related stress for 1 week prior to the study. The health of the mice was monitored by daily observation. Chlorinated water and irradiated food were provided ad libitum, and the animals were kept in a controlled light-dark cycle (12 h-12 h). Animal procedures were approved by the Institutional Animal Care and Use Committee at Chugai Pharmaceutical Co., Ltd..

### Cell lines and culture conditions

Nine human gastric cancer cell lines and two human colorectal cancer cell lines were used in the present study. MKN-45 and MKN-28 were purchased from Immuno-Biochemical Laboratories Co., Ltd. (Fujioka, Japan). NCI-N87, SCH, and HUVEC were obtained from the American Type Culture Collection (Rockville, MD, USA), the Japan Health Science Foundation (Osaka, Japan), and KURABO (Osaka, Japan), respectively. MKN-45 was maintained in TIL Media medium (Immuno-Biochemical Laboratories) supplemented with 10% (v/v) FBS, and MKN-28, NCI-N87, and SCH were maintained in RPMI-1640 supplemented with 10% (v/v) FBS at 37°C under 5% CO_2_. HUVEC was maintained in HuMedia-EG2 (KURABO) at 37°C under 5% CO_2_. Cell lines 4-1ST, SC-08-JCK, SC-09-JCK, SC-10-JCK and COL-16-JCK were purchased from the Central Institute for Experimental Animals (Yokohama, Japan). GXF97 and CXF280 were kindly provided by Prof. H. H. Fiebig (University of Freiberg, Freiberg, Germany). 4-1ST, SC-08-JCK, SC-09-JCK, SC-10-JCK, GXF97 and CXF280 were maintained in BALB/c-nu/nu mice by subcutaneous (sc) inoculation of pieces of the tumor tissue.

### In vivo tumor growth inhibition studies

Each mouse was inoculated sc into the right flank with either 5 × 10^6 ^cells/mouse of human gastric cancer cell line MKN-45, MKN-28, NCI-N87 or SCH, or an 8-mm^3 ^piece of 4-1ST, SC-08-JCK, SC-09-JCK, SC-10-JCK, GXF97 or CXF280 tumor tissue. Several weeks after tumor inoculation, the mice were randomly allocated to control and treatment groups. The administration of anticancer agents was started when tumor volumes reached approximately 0.2 to 0.3 cm^3^. To evaluate the antitumor activity of the test agents, tumor volume and body weight were measured twice a week. Tumor volumes (V) were estimated from the equation V = ab^2^/2, where a and b are tumor length and width, respectively. The percentage of tumor growth inhibition (TGI%) was calculated as described previously [10].

### Treatment of animals

Bevacizumab was administered intraperitoneally (ip) once a week for 3 weeks.

### Histological classification by hematoxylin-eosin staining

Xenograft tumor tissues were collected, formalin-fixed, and paraffin-embedded. Slide specimens were prepared by sectioning the tissue and staining with hematoxylin-eosin stain. The histological classification was determined according to the *Japanese Classification of Gastric Carcinoma *(13th Edition, June 1999, Japanese Gastric Cancer Association).

### IHC, and gene amplification of HER2

HER2 protein expression and HER2 gene amplification in tumors were examined by IHC using HercepTest^® ^and FISH using Pathvysion^® ^respectively as described previously [11].

### Measurement of microvessel density in tumor tissues

Microvessel density (MVD) in tumor tissue was evaluated immunohistochemically using a monoclonal anti-mouse CD31 antibody (rat anti-mouse CD31 monoclonal antibody, clone MEC 13.3; BD Biosciences, NJ, USA). Tumor samples were collected 24 h, 96 h or 21 days after bevacizumab administration. Immunohistochemical staining was performed as described previously [12]. MVD (%) was calculated from the ratio of the CD31-positive staining area to the total observation area in the viable region. Three to six fields per section (0.4856 mm^2 ^each) were randomly analyzed, excluding necrotic areas. Positive staining areas were calculated using imaging analysis software (Win Roof; Mitani Corporation, Fukui, Japan).

### Quantification of human or mouse VEGF and other angiogenic proteins in tumor tissues and mouse serum

Blood and tumor samples were taken when tumors had reached a volume of approximately 0.3 to 0.5 cm^3^. Blood serum was immediately retrieved and tumors were immediately frozen in liquid nitrogen and stored at -80°C. Tumor samples were homogenized in PBS containing 0.05% Tween 20 and centrifuged (4°C, 10,000 × g, 20 min.). The resultant supernatant was used for the assays. Human VEGF, placental growth factor (P*l*GF), interleukin-8 (IL-8) and basic fibroblast growth factor (bFGF) were quantified using Quantikine^® ^ELISA kits (R&D Systems, Minneapolis, MN, USA). Human VEGF165b was quantified using the DuoSet^® ^ELISA Development System (R&D Systems). Mouse VEGF was quantified using a Mouse VEGF Assay Kit (Immuno-Biochemical Laboratories). Total protein levels in the samples were quantified using a DC protein assay kit (BioRad Laboratories, Hercules, CA, USA).

### VEGF genotyping

DNA was extracted from the cultured cells. VEGF polymorphisms (-2578, -1154, -634 and 936 on VEGF genomic DNA) were genotyped with two fluorescent hybridization probes using LightCycler™ software. Briefly, the analysis is based on PCR amplification of the region surrounding the polymorphism sites, followed by slowly melting off the polymorphism-covering hybridized probe while continuously detecting the fluorescence. Melting off the hybridized probe from its target sequence causes the fluorescent signal to disappear, and this allows a narrow and reproducible estimation of the melting-point temperature. Subtle differences in melting points between a completely matched hybrid duplex and a duplex with a single nucleotide mismatch are detectable using LightCycler.

### HUVEC pVEGFR2 assay

Tumor samples were homogenized in HuMedia-EB2 (KURABO) and centrifuged (4°C, 10,000 × g, 20 min). The supernatant was retrieved and used as the assay medium. VEGF concentration in the samples was measured as described above. HUVEC was seeded at a density of 3 × 10^5 ^cells/well into 6-well plates in HuMedia-EG2 and incubated for 1 day at 37°C under 5% CO_2_. After that, the medium was changed to a serum-starved medium (HuMedia-EB2 containing 0.5% FBS). After overnight incubation, the medium was changed to an assay medium which had been pre-treated with bevacizumab or human IgG at 37°C for 30 min. After 5-min incubation at 37°C under 5% CO_2_, the plates were immediately placed on ice and the cells washed with PBS. The cells were then lysed with ice-cold lysis buffer #9 (R&D systems). Lysates were centrifuged at 14,000 × g for 20 min at 4°C. The pVEGFR2 was detected using western blot method and DuoSet IC^® ^ELISA Development System (R&D Systems). For western blot, antibodies against VEGFR2 and pVEGFR2 (Tyr1175) (#2479 and #2478 respectively, Cell Signaling Tschnology, Beverly, MA, USA) were used as the first antibodies. The proteins were detected by horseradish peroxidase-conjugated secondary antibodies (Santa Cruz Biotechnology, Santa Cruz, CA, USA) and visualized using ECL plus (GE Healthcare Life Science, Buckinghamshire, UK).

### Statistical analysis

The statistical analysis was carried out using a SAS preclinical package (SAS Institute, Inc., Tokyo, Japan).

## Results

### Sensitivity to bevacizumab in human gastric cancer xenograft models

**Figure 1:**
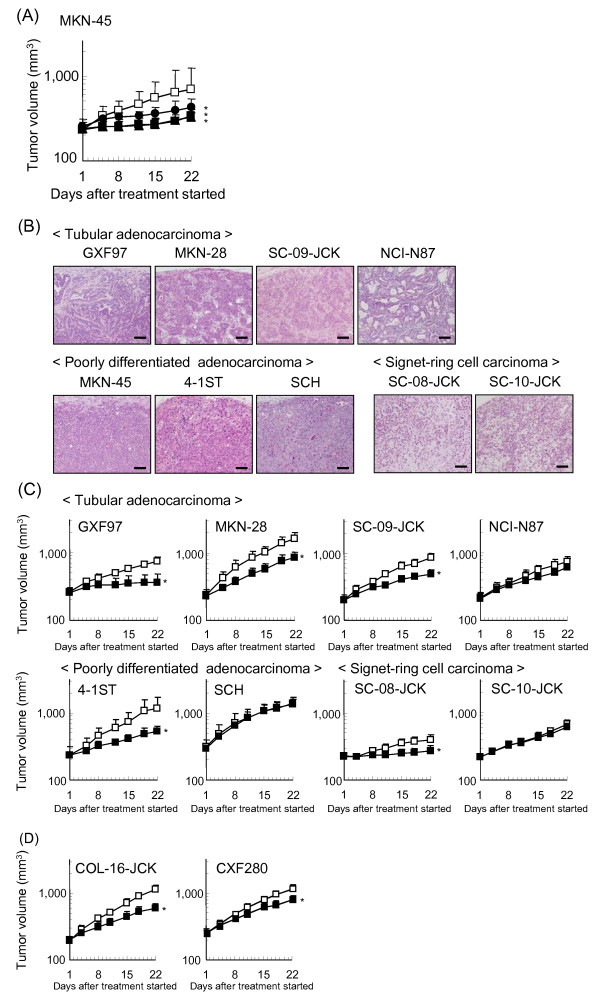
**Antitumor activity of bevacizumab as a single agent in human gastric cancer xenograft models**. (**A**) Mice bearing MKN-45 tumors were randomly divided into groups and HuIgG or bevacizumab (1.25, 5, 20 mg/kg) was administered intraperitoneally, once a week for 3 weeks. □: human IgG 20 mg/kg, ●: bevacizumab1.25 mg/kg, ■: bevacizumab 5 mg/kg, ▲: bevacizumab 20 mg/kg. (**B**) Differentiation degree of each tumor was determined by hematoxylin-eosin staining. (**C**) Antitumor activity of bevacizumab (5 mg/kg) was evaluated in various types of gastric cancer xenograft models: GXF97, MKN-28, NCI-N87, 4-1ST, SCH, SC-08-JCK, SC-09-JCK, SC-10-JCK. □: human IgG 20 mg/kg, ■: bevacizumab 5 mg/kg. (**D**) Antitumor activity of bevacizumab (5 mg/kg) was evaluated in two colorectal cancer xenograft models, COL-16-JCK and CXF280. □: human IgG 20 mg/kg, ■: bevacizumab 5 mg/kg. Data points are mean + SD of tumor volume (mm^3^). Statistically significant differences were shown as *: *P *< 0.05 vs. control group by Wilcoxon test. (*n *= 5-7/group).

We examined the antitumor activity of bevacizumab in the MKN-45 human gastric xenograft model. Bevacizumab showed significant antitumor activity against MKN-45 tumors at doses ranging from 1.25 mg/kg to 20 mg/kg (Figure 1A). On day 22 (21 days after starting treatment), tumor growth inhibition rates (TGI%) were 62%, 76%, and 79% at doses of 1.25, 5, and 20 mg/kg, respectively. The maximum effective dose was 5 mg/kg. Then we investigated 5 mg/kg of bevacizumab antitumor activity against human gastric cancer xenografts of various degrees of differentiation as shown in Figure 1B. Bevacizumab showed significant antitumor activity in GXF97, MKN-28, 4-1ST, SC-08-JCK and SC-09-JCK, with TGI% values on day 22 of 78%, 56%, 68%, 75%, and 55%, respectively. Meanwhile, bevacizumab did not show significant antitumor activity in NCI-N87, SCH and SC-10-JCK, with TGI% values on day 22 of 21%, 3% and 11%, respectively (Figure 1C). We also tested the antitumor activity of bevacizumab against two colorectal tumors, COL-16-JCK and CXF280, as the control because bevacizumab is approved for clinical use in colorectal cancers, and we found the TGI% to be 59% and 40%, respectively (Figure 1D). The degree of antitumor activity of bevacizumab in the gastric cancer models was comparable to that in the colorectal cancer models. No weight loss (> 20%) was observed for any of the doses tested in either model (data not shown).

### Histological types of tumor tissues

The degree of differentiation among the tumors was examined by using hematoxylin-eosin staining (Figure 1B). GXF97, MKN-28, SC-09-JCK, and NCI-N87 were categorized as tubular adenocarcinoma, known as a well differentiated type. MKN-45, 4-1ST and SCH were categorized as poorly-differentiated adenocarcinoma and all of them were solid-type. SC-08-JCK and SC-10-JCK were categorized as signet-ring cell carcinoma. There was no correlation between the efficacy of bevacizumab and the degree of differentiation of the tumors, and bevacizumab showed antitumor activity against every differentiation type of tumor we tested as shown in Figure 1.

### HER2 status of tumor tissues

HER2 status of tumor tissues which we examined bevasizumab sensitivity was shown in Table 1. There was also no relevancy between the efficacy of bevacizumab and the HER2 status, as shown by the observation that the HER2-positive tumor 4-1ST was sensitive to bevacizumab but HER2-positive tumors NCI-N87, and SCH were insensitive to bevacizumab.

**Table 1:** HER2 expression in gastrointestinal cancer cell lines

	HER2 status	
	
Cell line	IHC	FISH
NCI-N87	2+	8.4
4-1ST	3+	5.3
SCH	2+	2.0
SC-09-JCK	0	1.2
SC-10-JCK	0	1.2
MKN-45	0	1.1
MKN-28	0	1.0
SC-08-JCK	0	1.0
GXF97	0	0.9

HER2 protein expression and HER2 gene amplification in tumors were examined by IHC using HercepTest^® ^and FISH using Pathvysion^® ^respectively

### Expression of human VEGF

**Figure 2:**
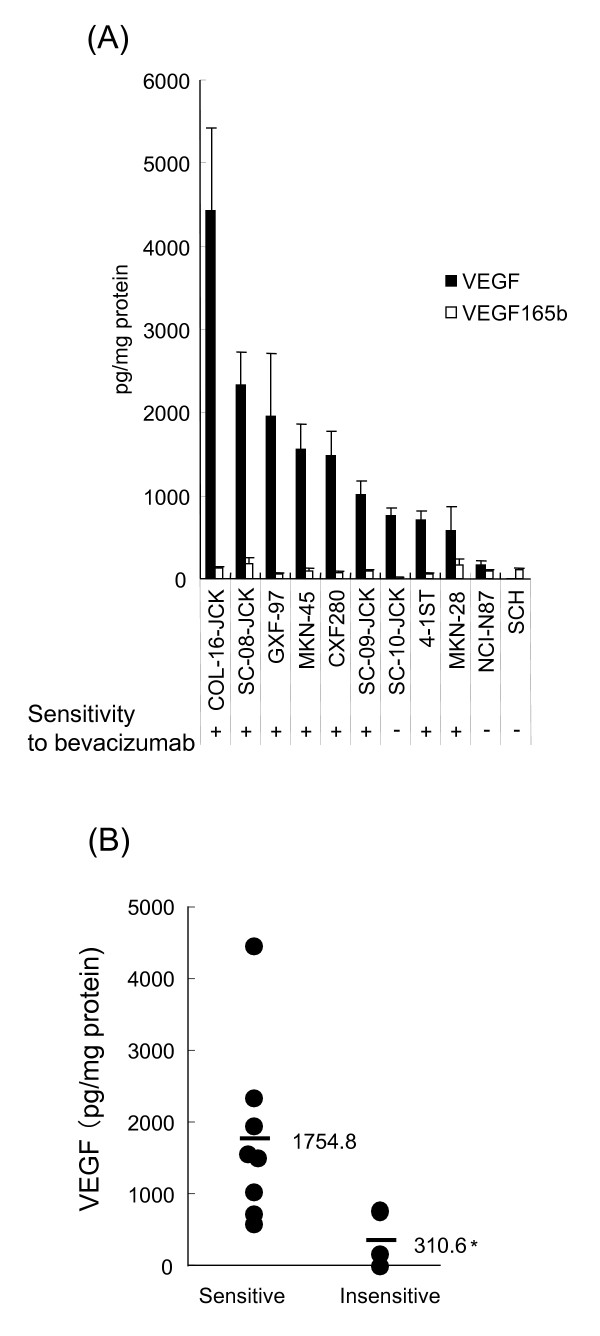
**Expression of human VEGF proteins in tumor tissues of gastric and colorectal cancer xenograft models**. (**A**) The level of human VEGF (black bar) and VEGF165b (open bar) protein in bevacizumab-sensitive and bevacizumab-insensitive tumor tissues was quantified by ELISA (*n *= 4/group). (**B**) The quantity of VEGF was compared between the two groups. The averages were shown as bars and numeric values. *: *P *< 0.05 vs. sensitive group by Wilcoxon test.

There is a possibility that the level of expression of human VEGF in tumor tissue is involved in the efficacy of bevacizumab, because bevacizumab is an anti-human VEGF monoclonal antibody. We quantified the expression level of human VEGF and VEGF165b in several gastric and colorectal tumor tissues (Figure 2A). We found that human VEGF was hardly expressed in bevacizumab-insensitive tumors SCH and NCI-N87 but, in the insensitive tumor SC-10-JCK, human VEGF was expressed. VEGF165b is reported to be an anti-angiogenic factor [13] and thus, could inhibit bevacizumab activity. However, it was not expressed to any extent in the tumors we investigated. Therefore, the effect of VEGF165b was considered to be negligible in the examined tumor models. Angiogenic human VEGF was more significantly expressed in bevacizumab-sensitive tumors compared with bevacizumab-insensitive tumors (Figure 2B, p = 0.0485). The correlation coefficient (r^2^) between TGI% and the log concentration of human VEGF per mg protein in tumor tissue was 0.5312. We also quantified the expression level of mouse VEGF in the tumors, but the correlation coefficient between TGI% and mouse tumor VEGF was not observed (r^2 ^= 0.0098). We also quantified human and mouse VEGF in serum, but the level of expression was below the limit of detection (< 31.25 pg/mL).

### Polymorphisms of VEGF

In clinical breast cancer patients, the efficacy of bevacizumab is reported to correlate to the status of VEGF polymorphism, the -2578A allele. Hence, we tried to examine the correlation of VEGF polymorphisms and efficacy, but statistical analysis could not be done because the A/A allele was only observed in the MKN-45 tumor. The level of VEGF in MKN-45 was not high, even though the -2578 allele exists in the VEGF promoter (Table 2).

**Table 2:** VEGF polymorphisms in gastrointestinal cancer cell lines

	Genotype			
	
	Polymorphic site			
Cell line	-2578	-1154	-634	936
MKN-45	A/A	A/A	G/G	T/T
4-1ST	C/A	G/A	G/G	C/C
GXF97	C/A	G/A	C/G	C/C
MKN-28	C/C	G/A	C/C	C/C
SC-09-JCK	C/C	G/A	G/G	C/C
SC-08-JCK	C/C	G/A	C/C	T/T
NCI-N87	C/A	G/A	G/G	C/C
SC-10-JCK	C/A	G/A	G/G	C/T
SCH	C/C	G/A	C/G	C/C

VEGF polymorphisms (-2578, -1154, -634 and 936 on VEGF genomic DNA) were examined by using LightCycler™

### Bioactivity of VEGF in SC-10-JCK

**Figure 3:**
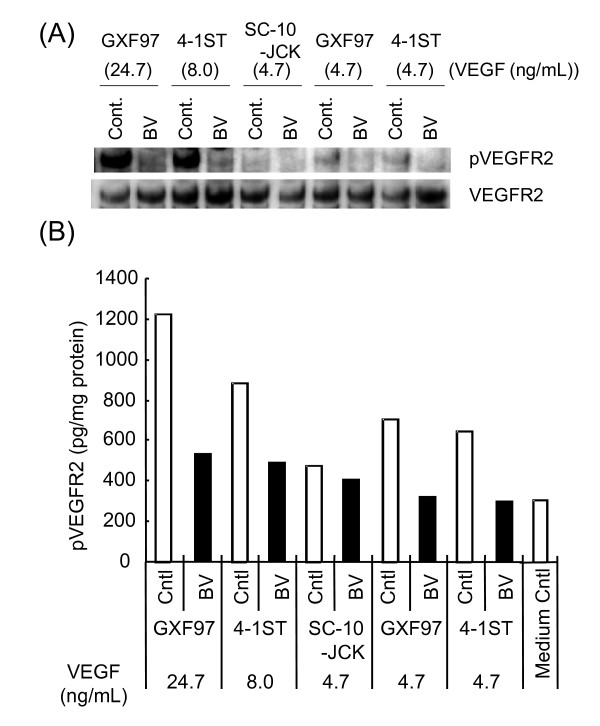
**Differences in the bioactivity of human VEGF in bevacizumab-sensitive tumors and bevacizumab-insensitive tumor SC-10-JCK**. The bioactivity of VEGF in tumors was determined by the VEGFR2 phosphorylation of HUVEC treated with tumor tissue homogenate administered with bevacizumab or human IgG using western blot (**A**) and ELISA (**B**). Each VEGF level in the homogenate was shown in Figure 3A and B.

We examined whether the VEGF expressed in SC-10-JCK was bioactive. Tumor tissue lysates of two bevacizumab-sensitive models, GXF97 (high VEGF expression) and 4-1ST (VEGF level equivalent to SC-10-JCK), and one bevacizumab-insensitive model, SC-10-JCK, with or without bevacizumab, were added to HUVEC, and the phosphorylation level of VEGFR2 in HUVEC was tested by western blot (Figure 3A). VEGF levels of the lysates of GXF97, 4-1ST and SC-10-JCK were 24.7, 8.0 and 4.7 ng/mL respectively. VEGF derived from bevacizumab-sensitive tumors (GXF97 and 4-1ST) phosphorylated VEGFR2. The VEGF diluted to 4.7 ng/mL (a concentration equal to SC-10-JCK-derived VEGF) still phosphorylated VEGFR2. All phosphorylation of VEGFR2 by VEGF from bevacizumab-sensitive tumors was reduced by bevacizumab. On the other hand, SC-10-JCK-derived VEGF did not phosphorylate VEGFR2 both in western blot and ELISA (Figure 3B). These results suggest that the VEGF in SC-10-JCK was not bioactive.

### Anti-angiogenic activity of bevacizumab

**Figure 4:**
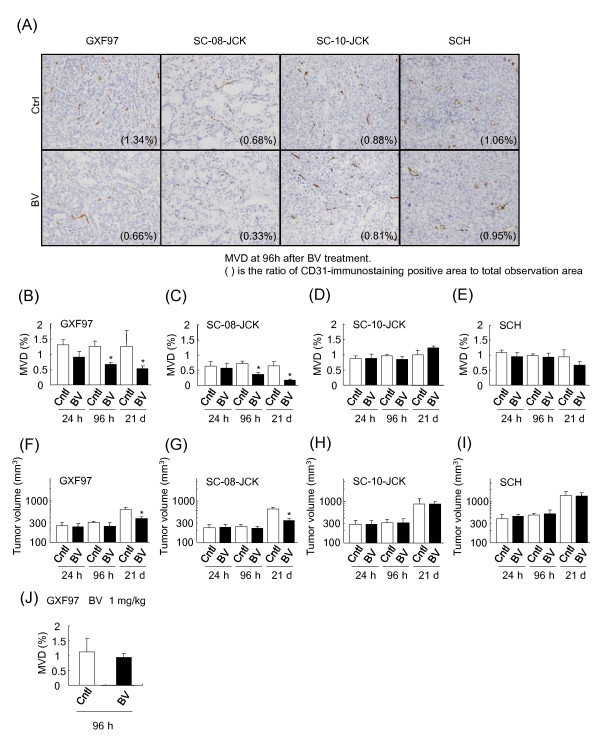
**Change in MVD after bevacizumab treatment**. (**A**) CD31 immunostaining in tumor tissue at 96 h after treated with HuIgG (Ctrl) and bevacizumab (BV) in GXF97, SC-08-JCK, SC-10-JCK and SCH models. (**B**-**E**) The MVD in tumor tissue was determined by calculating the ratio of CD31-immunostaining positive area to the total observation area at 24 h, 96 h and 21 days (21 d) after bevacizumab treatment (*n *= 3-6/group). (**F**-**I**) Tumor volumes were measured on the day of MVD evaluation. (**J**) MVD after treatment of bevacizumab at 1 mg/kg was evaluated in GXF97 (*n *= 4). *: *P *< 0.05 vs. control group by Wilcoxon test.

We also examined changes in MVD in bevacizumab-sensitive and -insensitive tumors after bevacizumab treatment using CD31-staining (Figure 4A). In the bevacizumab-sensitive tumor models, GXF97 and SC-08-JCK, a significant decrease of MVD was observed at 96 h after single-treatment of bevacizumab and at 21 days after the 3-week treatment (Figure 4B and 4C). On the other hand, MVD had not changed at any time point after bevacizumab treatment in the bevacizumab-insensitive tumor models, SC-10-JCK and SCH (Figure 4D and 4E). The decrease of MVD was corresponded to the antitumor activity of bevacizumab, but was detected earlier than the decrease of tumor volume (Figure 4F-I). We also examined the change in MVD in the GXF97 tumor samples at the lower dose (1 mg/kg), at which bevacizumab had not shown efficacy on day 22 (Figure 4J). In correlation with the efficacy of bevacizumab, MVD had not changed in GXF97 tumor tissue 96 h after bevacizumab treatment at the low dose. Therefore we considered that bevacizumab showed antitumor activity by inhibiting tumor angiogenesis.

### Other angiogenic factors as the bevacizumab resistance factor in tumor tissues

**Figure 5:**
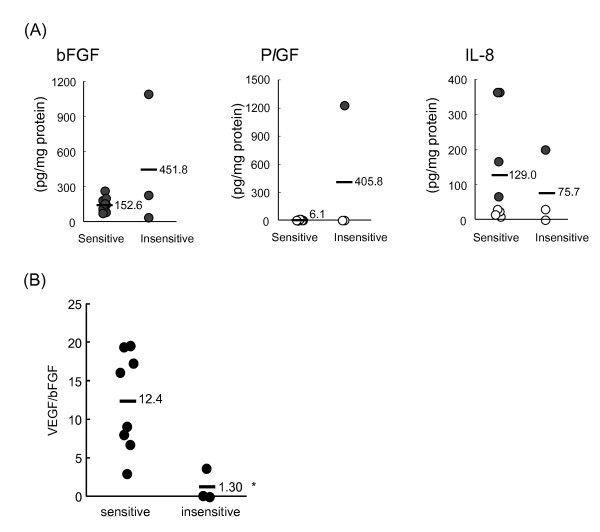
**Expression of human bFGF protein in tumor tissues of gastric and colorectal cancer xenograft models**. (**A**) The level of human bFGF, PlGF, and IL-8 proteins in tumor tissues sensitive (9 models) or insensitive (3 models) to bevacizumab was quantified by ELISA (*n *= 4). Open circle means less than the detection limit of ELISAs. The averages were shown as bars and numeric values. (**B**) The ratio of VEGF to bFGF was compared between the two groups. *: *P *< 0.05 vs. sensitive group by Wilcoxon test.

The MVD results after bevacizumab treatment suggested that angiogenic factors other than VEGF are involved in angiogenesis in bevacizumab-insensitive tumors. Therefore, we investigated the expression levels of angiogenic factors bFGF, IL-8 and P*l*GF in tumor tissues, all of which are reported to be angiogenic factors for tumors (Table 3). Each one as a single factor did not significantly correlate with bevacizumab efficacy (Figure 5A). However, the VEGF/bFGF ratio in bevacizumab-insensitive tumors was significantly lower than that in bevacizumab-sensitive tumors (1.30% vs. 12.4%, Figure 5B, p = 0.0242).

**Table 3:** Expression of angiogenic factors in gastrointestinal cancer cell lines

Cell line	Sensitivity to bevacizumab	VEGF	PlGF	IL-8	bFGF
COL-16-JCK	+	4430 ± 991	< 15.6	360 ± 171	268 ± 75.3
SC-08-JCK	+	2330 ± 402	< 15.6	< 31.3	145 ± 25.9
GXF97	+	1950 ± 753	< 15.6	362 ± 134	107 ± 63.6
MKN-45	+	1560 ± 310	< 15.6	65.9 ± 9.38	183 ± 43.0
CXF280	+	1480 ± 290	< 15.6	< 31.3	77.6 ± 16.5
SC-09-JCK	+	1020 ± 163	< 15.6	165 ± 60.3	151 ± 13.3
4-1ST	+	706 ± 106	< 15.6	< 31.3	87.7 ± 5.47
MKN-28	+	577 ± 285	16.4 ± 8.22	< 31.3	200 ± 27.8
SC-10-JCK	-	760 ± 80.2	< 15.6	198 ± 96.5	231 ± 105
NCI-N87	-	171 ± 52.5	< 15.6	34.4 ± 10.8	1090 ± 328
SCH	-	< 31.3	1210 ± 205	< 31.3	38.4 ± 18.6

Mean ± SD (pg/mg protein)

The expression of human VEGF, PlGF, IL-8 and bFGF in tumor tissues were examined by ELISA

## Discussion

Gastric cancer has long been classified according to degree of differentiation and histopathologic type. Recently, HER2 expression has been included as a classification method in response to the positive results of a phase III study showing that addition of trastuzumab to a combination of capecitabine (or 5FU) and CDDP in patients with HER2-overexpressing gastric cancers prolonged overall survival [14]. In light of this, we attempted to clarify the types of gastric cancers for which bevacizumab would be effective. We used human cancer xenograft models developed by inoculating human gastric cancer cells into T-cell-deficient mice to investigate the efficacy of bevacizumab. Human VEGF secreted from human cancer cells is known to have an effect on mouse endothelial cells and results in angiogenesis in xenograft tumor tissues. In MKN-45, bevacizumab showed significant antitumor activity, although the dose dependency was not strong. A low dose of bevacizumab (1.25 mg/kg) was sufficient to produce antitumor activity. We examined the antitumor activity of bevacizumab in 9 human gastric cancer xenograft models of various degrees of differentiation, tumor types, and HER2 expression. However, the sensitivity of the gastric cancer models to bevacizumab was found to be unrelated to the histological type or the HER2 status of the tumors. The antitumor activity of bevacizumab in the gastric cancer xenograft models was similar to that observed in colorectal cancer [15], for which bevacizumab has shown survival and clinical benefit.

Next, we examined other biomarker candidates of bevacizumab efficacy using sensitive and insensitive gastric tumor models. Because VEGF is the molecular target of bevacizumab, the tumor level of VEGF is thought to influence the sensitivity to bevacizumab. Tumor VEGF levels were significantly lower in the xenograft models insensitive to bevacizumab than in the sensitive models; however, mouse tumor VEGF showed no differences between sensitive and insensitive tumors. Human and mouse blood VEGF were lower than the detection limit in these models, and thus they were of little concern. In the SC-10-JCK model, in spite of VEGF expression, bevacizumab did not exhibit efficacy. To clarify the role of VEGF as a biomarker candidate of bevacizumab sensitivity, we investigated subtypes of VEGF in more detail. Various isoforms of VEGF are known such as the reportedly anti-angiogenic VEGF, VEGF165b [13]. VEGF165b binds to bevacizumab [16]. In our study, VEGF165b exhibited 100% cross-reactivity to VEGF ELISA (data not shown) so we thought that VEGF165b expression would be high in SC-10-JCK. However, the level of VEGF165b was low in the SC-10-JCK model. Additionally, VEGF165b expression was low in all of the tumor tissues we examined. In the gastrointestinal models tested, VEGF165b is not a concern. Because bevacizumab binds to all angiogenic isoforms of VEGF, such as VEGF121, VEGF165, and VEGF189 [17], identification of these isoforms is not necessary. In clinical breast cancer treatment, polymorphisms of VEGF have been found to correlate with the efficacy of the bevacizumab and paclitaxel combination [18]. The survival time of patients with the 2578 AA allele or 1154 A allele of VEGF was longer than that of others. However, VEGF 2578 and 1154 of SC-10-JCK were CA and GA, respectively, and the type of allele was the same as with the sensitive tumors 4-1ST and GXF97. The VEGF allele did not distinguish sensitivity to bevacizumab in these models. Next, to clarify whether the VEGF of SC-10-JCK had biological activity, the effect of a tumor homogenate of SC-10-JCK on the induction of VEGFR phosphorylation on HUVEC was examined. The tumor homogenate of SC-10-JCK did not have phosphorylation activity in contrast to that of 4-1ST or GXF97. Furthermore, the MVD in the SC-10-JCK xenograft model treated with bevacizumab was not reduced, indicating that the VEGF of SC-10-JCK did not show angiogenic activity. These results suggest the possibility of existence of non-active VEGF in tumors.

We attempted to clarify both the biomarker for and the mechanisms of bevacizumab. The inhibition of MVD in tumor tissue is reported to be a major mechanism of bevacizumab efficacy, and so we compared MVD inhibition with efficacy in bevacizumab-sensitive and -insensitive tumor models. The existence of MVD inhibition corresponded with antitumor efficacy. In the present study, the MVD inhibition prior to antitumor efficacy was shown, and MVD inhibition was found to be a mechanism of bevacizumab efficacy. Our results indicated that other angiogenic factors except for VEGF are involved in biomarkers for bevacizumab resistance. We examined tumor bFGF, IL-8 and P*l*GF, all of which are reported to be angiogenic factors for tumors [19-21]. When we compared the ratio of bFGF to VEGF with bevacizumab efficacy to clarify the extent of the role of VEGF in angiogenesis, the ratio of VEGF to bFGF in bevacizumab-insensitive tumors was clearly lower than that in bevacizumab-sensitive tumors. It is thought that an intimate cross-talk of bFGF and VEGF family is exist during angiogenesis and bFGF increases neuroplin-1, a co-receptor for VEGF, expression in human vascular smooth muscle cell that in turn enhance the effect of VEGF on cell migration and promote neovascularizaion [22]. In the present study, we did not examine the bioactivity of bFGF in the bevacizumab insensitive tumors. It is necessary to investigate the effect of bFGF on VEGF-dependent angiogenesis and on its inhibition by bevacizumab in the future. Neither VEGF/PlGF nor VEGF/IL-8 showed significant results (data not shown). P*l*GF has been reported to form a homodimer or heterodimer with VEGF and bind the resulting VEGFR in angiogenesis [23]. However, the cell lines we examined did not express P*l*GF, except for SCH, so we examined the relationship between P*l*GF and bevacizumab in SCH in vitro. HUVEC growth induction with a P*l*GF/VEGF heterodimer was inhibited by bevacizumab (data not shown), suggesting that the number of heterodimers of P*l*GF and VEGF would also be a biomarker for bevacizumab sensitivity. Further examination is needed. P*l*GF homodimers did not show HUVEC growth induction, and we did not examine the effect of bevacizumab on HUVEC growth.

Some reports have indicated that bevacizumab exhibits direct cell growth inhibition against tumor cells [24,25]. However, in the gastric cancer cell lines we examined, no direct antitumor activity was observed in spite of the growth inhibition shown by bevacizumab in the xenograft model of the same cancer cell line (data not shown).

## Conclusions

In the present study of gastric cancer treatment, we found that levels of VEGF and the VEGF/bFGF ratio have a close correlation with sensitivity to bevacizumab in the tested cell lines. There was no correlation between sensitivity to bevacizumab and the histological classification according to the degree of differentiation or HER2 status of the tumor cells. Clinical evidence is expected.

## Competing interests

The authors declare that they have no competing interests.

## Authors' contributions

YYK designed experiments, performed experiments and statistical analysis, interpreted results and drafted manuscript. KFO established the study concept and conducted critical revision to manuscript. KY, MK, MY and HY performed experiments. KM performed peer reviewing the final draft. All authors have read and approved the final manuscript.

## Pre-publication history

The pre-publication history for this paper can be accessed here:

http://www.biomedcentral.com/1471-2407/12/37/prepub
